# Supplementary material for: Decoding a neural circuit controlling global animal state in C. elegans
Source: eLife. 2015 Mar 11;4:e04241. doi: 10.7554/eLife.04241 (PMC4440410; doi:10.7554/eLife.04241)
Supplement: Supplementary file 5. — Lists of strains and constructs. DOI: http://dx.doi.org/10.7554/eLife.04241.024 [file elife04241s005.docx]

**Laurent Soltesz et al.**

**Supplementary File 5**

**Strains list**

AQ2460 *ljIs124[Pgpa-13::FLPase; Psra-6::FTF-ChR2-YFP]*

AX1006 *lon-2(e678) npr-1(ad609); ttx-3(ks5)*

AX1107 *npr-1(ky13) flp-21(pk1601)*

AX1112 *npr-1(g320) flp-21(pk1601)*

AX1129 *flp-21(pk1601)*

AX1232 *npr-1(ad609);gcy-35(ok769); dbEx[pflp-8::gcy-35 cDNA-SL2-GFP]*

AX1864 *npr-1(ad609); lin-15(n765ts); dbEx[Pgcy-32::YC3.60; lin-15(+)]*

AX2323 *npr-1(ad609); hpIs190[Pnmr-1::D3cpv]*

AX2389 *ocr-2(ak47); npr-1(ad609); dbEx[psra-6::genomic ocr-2]*

AX2391 *ocr-2(ak47); npr-1(ad609); dbEx[psrh-220::genomic ocr-2]*

AX2393 *ocr-2(ak47); npr-1(ad609); dbEx[psrh-142::genomic ocr-2]*

AX3100 *npr-1(ad609);lite-1(ce314); dbEx601[pflp-8::YC3.60; lin-15(+)]*

AX3101 *npr-1(ad609);lite-1(ce314); unc-64(e246); dbEx601[pflp-8::YC3.60; lin-15(+)]*

AX3144 *npr-1(ad609); dbEx608[Pgcy-28::NpHR-mCherry; ccRFP]*

AX3167 *npr-1(ad609); dbEx[Pncs-1::Cre; Pflp-21::LoxP-STOP-LoxP-ChR2-mCitrine; ccRFP]*

AX3176 *npr-1(ad609); dbEx[Pncs-1::Cre; Pflp-21::LoxP-STOP-LoxP-YC2.60; ccRFP]*

AX3208 *npr-1(ad609); dbEx[Pins-1::YC2.60]*

AX3209 *npr-1(ad609); dbEx[Pgcy-28::YC3.60]*

AX3428 *npr-1(ad609); hpIs179[Psra-11::D3cpv]*

AX3431 *npr-1(ad609); hpIs157[Pglr-1::YC3.60]*

AX3435 *npr-1(ad609);ocr-2(ak47); dbEx[Pncs-1::Cre; Pflp-21::LoxP-STOP-LoxP-YC2.60; ccRFP]*

AX3437 *npr-1(ad609);gcy-35 (ok769);dbEx[Pncs-1::Cre; Pflp-21::LoxP-STOP-LoxP-YC2.60; ccRFP]*

AX3438 *npr-1(ad609);unc-64(e246); dbEx[Pncs-1::Cre; Pflp-21::LoxP-STOP-LoxP-YC2.60; ccRFP]*

AX3475 *npr-1(ad609); lite-1(ce314); hpIs171[Pacr-2::D3cpv]*

AX3478 *npr-1(ad609); hpIs157[Pglr-1::YC3.60]; dbEx608[Pgcy-28::NpHR-mCherry; ccRFP]*

AX3935 *dbEx[Pncs-1::Cre; Pflp-21::LoxP-STOP-LoxP-ChR2-mCitrine; ccRFP]*

AX3936 *npr-1(ad609); ocr-2(ak47); dbEx[Pncs-1::Cre; Pflp-21::LoxP-STOP-LoxP-ChR2-mCitrine; ccRFP]*

AX3937 *npr-1(ad609); ljIs124[Pgpa-13::FLPase; Psra-6::FTF-ChR2-YFP]*

AX3938 *npr-1(ad609); ocr-2(ak47); ljIs124[Pgpa-13::FLPase; Psra-6::FRT-STOP-FRT-ChR2-YFP]*

AX3939 *npr-1(ad609); ocr-2(ak47); dbEx608[Pgcy-28::NpHR-mCherry; ccRFP]*

AX3975 *npr-1(ad609) dbEx[Pglr-5::Cre(co); Psra-11::LoxP-STOP-LoxP-NpHR-mCherry; ccRFP]*

AX4018 *npr-1(ad609); dbEx[psra-9::YC3.60; ccRFP]*

AX4021 *npr-1(ad609); gcy-35(ok769) dbEx[psra-9::YC3.60; ccRFP]*

AX4175 *lon-2(e678); npr-1(ad609); ttx-3(ks5); dbIs[Pins-1::Caspase ;ccGFP]*

AX4176 *npr-1(ad609); dbIs[Pins-1::Caspase; ccGFP]*

AX4188 *npr-1(ad609); qrIs2[Psra-9::mCaspase-1; ccGFP]*

AX4189 *npr-1(ad609); qaIs2241[Pgcy-32::EGL-1; Pgcy-35::GFP; lin-15(+)]; dbEx[Pncs-1::Cre; Pflp-21::LoxP-STOP-LoxP-ChR2-mCitrine; ccRFP]*

AX4190 *npr-1(ad609); qrIs2[Psra-9::mCaspase-1; ccGFP]; dbEx[Pncs-1::Cre; Pflp-21::LoxP-STOP-LoxP-ChR2-mCitrine; ccRFP]*

AX4233 *npr-1(ad609); dbEx[Pncs-1::Cre; Pflp-21::LoxP-STOP-LoxP-EGL-21RNAiSense-SL2-RFP; Pflp-21::LoxP-STOP-LoxP-EGL-21RNAiAntisense-SL2-RFP; HygR; ccRFP]*

AX4236 *npr-1(ad609); ynIs72 [Pflp-1::GFP]*

AX4281 *npr-1(ad609); dbEx[Pgpa-11::miniSOG-SL2-RFP]; dbEx[Pncs-1::Cre; Pflp-21::LoxP-STOP-LoxP-ChR2-mCitrine; ccRFP]*

AX4282 *npr-1(ad609); ynIs80 [Pflp-21(2kb)::GFP]*

AX4284 *npr-1;gcy-35(ok769); ynIs80 [Pflp-21(2kb)::GFP]*

AX4286 *ocr-2(ak47); npr-1(ad609); ynIs80 [Pflp-21(2kb)::GFP]*

AX4288 *ynIs80 [Pflp-21(2kb)::GFP]*

AX4290 *npr-1(ad609); ynIs49[Pflp-5::GFP]*

AX4292 *gcy-35(ok769); npr-1(ad609); Is[Pflp-5::GFP]*

AX4294 *ocr-2(ak47); npr-1(ad609); Is[Pflp-5::GFP]*

AX4296 *Is[Pflp-5::GFP]*

AX4298 *npr-1(ad609); ynIs40 [Pflp-11::GFP]*

AX4301 *npr-1(ad609); gcy-35 (ok769); ynIs40 [Pflp-11::GFP]*

AX4303 *npr-1(ad609); ocr2 (ak47); ynIs40 [Pflp-11::GFP]*

AX4304 *ynIs40 [Pflp-11::GFP]*

AX4310 *ynIs72 [Pflp-1::GFP]*

AX5331 *npr-1(ad609); dbEx[Pncs-1::Cre;  Pflp-21::LoxP-STOP-LoxP-FLP-21RNAiSense-SL2-RFP; Pflp-21::LoxP-STOP-LoxP-FLP-21RNAiAntisense-SL2-RFP; HygR; ccRFP]*

AX5721 *ocr-2(ak47); npr-1(ad609); dbEx[Psrh-220::genomic OCR-2; ccGFP]*

AX5722 *ocr-2(ak47); npr-1(ad609); dbEx[Psra-6::genomic OCR-2; ccGFP]*

AX5723 *ocr-2(ak47); npr-1(ad609); dbEx[Psrh-220::genomic OCR-2; ccGFP]; dbEx[Pgpa-11::TeTx-SL2-mCherry; ccRFP]*

AX5724 *ocr-2(ak47); npr-1(ad609); dbEx[Psra-6::genomic OCR-2; ccGFP]; dbEx[Pgpa-11::TeTx-SL2-mCherry; ccRFP]*

AX5725 *npr-1(ad609); dbEx[Pgpa-11::TeTx-SL2-mCherry; ccRFP]*

AX5726 *npr-1(ad609); dbEx[Pgcy-32::TeTx-SL2-RFP]*

AX5742 *npr-1(ad609); ynIs49[Pflp-5::GFP]; dbEx [Pflp-5::LoxP-STOP-LoxP::tetanus toxin-SL2-mCherry; Pncs-1::CRE]*

AX5743 *npr-1(ad609); dbIs [Pflp-21 4kb::GFP]*

AX5744 *npr-1(ad609); gcy-35 (ok769); dbIs [Pflp-21 4kb::GFP]*

AX5745 *npr-1(ad609); ocr-2(ak47); dbIs [Pflp-21 4kb::GFP]*

AX5746 *dbIs [Pflp-21 4kb::GFP]*

AX5747 *npr-1(ad609); dbEx [Pgpa-11::tetanus toxin::SL2mCherry; ccRFP]; dbIs [Pflp-21 4kb::GFP]*

AX5748 *npr-1(ad609); ynIs64[Pflp-17::GFP]*

AX5749 *npr-1(ad609); ynIs21[Pflp-3::GFP]*

AX5750 *npr-1(ad609); dbEx[Psra-6::YC3.60]*

AX5751 *npr-1(ad609); gcy-35(ok769); dbEx[Psra-6::YC3.60]*

AX5752 *dbEx[Pncs-1::Cre; Pflp-21::LoxP-STOP-LoxP-YC2.60; ccRFP]*

AX5753 *npr-1(ad609); dbEx[pttx-3::ChR2; ccRFP]*

AX5758 *npr-1(ad609); dbEx [Pgcy-32::tetanus toxin::SL2mCherry; ccRFP]; ynIs40 [Pflp-11::GFP]*

AX5759 *npr-1(ad609); myEx731[Ppdf-1 3kb::rfp + Punc-122::gfp]*

AX5775 *npr-1(ad609); ynIs40[Pflp-11:GFP]; dbEx[pgcy-32::egl-2(gf)-SL2-mcherry; ccRFP]*

AX5776 *lon-2(e678) npr-1(ad609); ttx-3(mg158)*

BOL171 *npr-1(ad609); lin-15(n765); dbEx[Pncs-1::Cre; Pflp-21::LoxP-STOP-LoxP-NPR-1(215V)-SL2-GFP; lin-15(+)]*

QR54 *qrIs2[Psra-9::mCaspase-1; ccGFP]*

RB982 *flp-21(ok889)*

ZM5091 *hpIs190[Pnmr-1::D3cpv]*

**Constructs list**

*Pncs-1*::*Cre*

*Pflp-21::LoxP-STOP-LoxP-YC2.60*

*Pflp-21::LoxP-Stop-LoxP-ChR2-mctirine*

*Pflp-21::LoxP-STOP-LoxP-NPR-1(215V)-SL2-GFP*

*Pgpa-11::miniSOG-SL2-RFP.*

*Pgcy-32::TeTx-SL2-RFP]*

*Pgcy-32::egl-2(gf)::mcherry*

*Pgpa-13::FLPase* (Y. Tanizawa, personal com)

*Psra-6::FRT-STOP-FRT-ChR2-YFP]* (Y. Tanizawa, personal com)

*Pglr-5::Cre(co)*

*Psra-11::LoxP-STOP-LoxP-NpHR-mCherry*

*Pgcy-28::NpHR-mCherry*

*Pflp-8::YC3.60*

*Pflp-8::gcy-35 cDNA-SL2-GFP*

*Pgcy-32::YC3.60*

*Pins-1::YC2.60*

*Pncs-1::Cre; Pflp-21::LoxP-STOP-LoxP-ChR2-mCitrine*

*Pgcy-28::YC3.60]*

*Pgpa-11::TeTx-SL2-mCherry*

*Pflp-5::LoxP-STOP-LoxP::tetanus toxin-SL2-mCherry*

*Pflp-21 4kb::GFP*

*gpa-11::tetanus toxin::SL2mCherry*

*Psra-6::YC3.60*

*psra-9::YC3.60*
